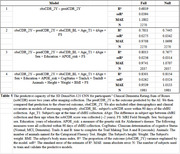# Predicting Future Clinical Decline with Deep Learning, Neuroimaging, and Demographic Data

**DOI:** 10.1002/alz70856_103816

**Published:** 2025-12-26

**Authors:** Christopher J Patterson, Nikhil J Dhinagar, Emma J Gleave, Sophia I Thomopoulos, Tamoghna Chattopadhyay, Paul M. Thompson

**Affiliations:** ^1^ Imaging Genetics Center, Mark and Mary Stevens Neuroimaging and Informatics Institute, Keck School of Medicine, University of Southern California, Marina Del Rey, CA, USA; ^2^ Imaging Genetics Center, Mark and Mary Stevens Neuroimaging & Informatics Institute, University of Southern California, Marina del Rey, CA, USA

## Abstract

**Background:**

Each year, approximately 15% of elderly individuals with mild cognitive impairment (MCI) progress to Alzheimer's disease (AD) or related dementias. Predicting this progression is crucial for informing treatment decisions, identifying protective factors, and modeling treatment effects in clinical trials. Here we trained and tested a 3D convolutional neural network (CNN) to forecast future decline (over two years) in the Clinical Dementia Rating scale sum of boxes score (sobCDR).

**Methods:**

We analyzed data from three independent, publicly‐available cohorts: the (1) Alzheimer's Disease Neuroimaging Initiative (ADNI; *n* = 1,136), (2) Open Access Series of Imaging Studies (OASIS‐3; *n* = 241), and (3) National Alzheimer's Coordinating Center (NACC; *n* = 942). Participants with a 3D T1‐weighted brain MRI, baseline age, sex, & BMI measures, baseline sobCDR (within 90 days of imaging), and a follow‐up sobCDR score (1.75‐2.25 years after baseline) were included.

For evaluation, datasets were partitioned into five independent, cross‐validation folds with balanced sex, age at imaging, and change in sobCDR score (scale 0: no impairment – 18: severe impairment) between baseline and 2 years.

A 3D DenseNet‐121 CNN was used to predict the sobCDR score 2 years after baseline, first by using only the T1‐weighted MRI. This predicted value (predCDR_2Y) was then included in four linear mixed‐effects models (LMEs) to predict the observed sobCDR at two years from baseline (obsCDR_2Y) that included additional demographic covariates, scanner manufacturer and participant cohort random effects (Table 1).

For each linear model, we ran a corresponding null model with all the same covariates, but without the predCDR_2Y variable, to determine the added predictive value of the imaging. From the five cross‐validation folds, we calculated the Pooled, out‐of‐sample, R2 and its standard error (SE), adjusting for the number of explanatory variables in each model.

**Results:**

All predictive models predicted follow‐up sobCDR with mean absolute error of around 1 point; adding the image‐derived prediction improved accuracy (Table 1).

**Conclusion:**

Deep learning shows promise for enhancing prognostic models; future work will include additional deep learning methods, more diverse neuroimaging data (amyloid and tau PET and diffusion MRI), and alternative data fusion methods to integrate tabular and neuroimaging data.